# A Novel Method for the Measurement of the Vaginal Wall Thickness by Transvaginal Ultrasound: A Study of Inter- and Intra-Observer Reliability

**DOI:** 10.3390/medicina60030370

**Published:** 2024-02-22

**Authors:** Sara Bosio, Marta Barba, Annalisa Vigna, Alice Cola, Desirèe De Vicari, Clarissa Costa, Silvia Volontè, Matteo Frigerio

**Affiliations:** 1 Department of Gynecology, Chiari HosSST Franciacorta, 25032 Chiari, Italy; sarabos2004@hotmail.it; 2 Department of Gynecology, IRCCS San Gerardo dei Tintori, University of Milano-Bicocca, 20900 Monza, Italy; m.barba8792@gmail.com (M.B.); alice.cola1@gmail.com (A.C.); d.devicari@campus.unimib.it (D.D.V.); c.costa14@campus.unimib.it (C.C.); s.volonte6@campus.unimib.it (S.V.); 3 Department of Gynecology, IRCCS Policlinico San Martino, University of Genova, 16132 Genova, Italy; annalisa.vigna.92@gmail.com

**Keywords:** ultrasound, vaginal wall thickness, genitourinary syndrome of menopause, pelvic floor disorders

## Abstract

*Background and Objectives*: A consensus regarding the optimal sonographic technique for measuring vaginal wall thickness (VWT) is still absent in the literature. This study aims to validate a new method for measuring VWT using a biplanar transvaginal ultrasound probe and assess both its intra-operator and inter-operator reproducibility. *Material and Methods*: This prospective study included patients with genitourinary syndrome of menopause-related symptoms. Women were scanned using a BK Medical Flex Focus 400 with the 65 × 5.5 mm linear longitudinal transducer of an endovaginal biplanar probe (BK Medical probe 8848, BK Ultrasound, Peabody, MA, USA). Vaginal wall thickness (VWT) measurements were acquired from the anterior and posterior vaginal wall at three levels. *Results*: An inter-observer analysis revealed good consistency between operators at every anatomical site, and the intra-class coefficient ranged from 0.931 to 0.987, indicating high reliability. An intra-observer analysis demonstrated robust consistency in vaginal wall thickness measurements, with an intra-class coefficient exceeding 0.9 for all anatomical sites. *Conclusions*: The measurement of vaginal wall thickness performed by transvaginal biplanar ultrasound was easy and demonstrated good intra- and inter-operator reliability.

## 1. Introduction

Genitourinary syndrome of menopause (GSM) and vulvovaginal atrophy (VVA) represent frequently reported menopause-related clinical entities [1]. During the fertile years, the female genital organ’s tropism is due to the estrogenic hormonal stimulus. With aging and falling estrogen levels, the estrogen-sensitive tissue becomes thinner, with losses in blood vessels, collagen, and elastin fibers, and changes in the quality and quantity of secretions [2]. Since the mucosal layer of the vagina is primarily composed of an estrogen-sensitive stratified squamous epithelium, the thickness of the vaginal wall decreases after menopause [3]. The reduced thickness of the vaginal epithelium can lead to vaginal atrophy, a condition marked by diverse symptoms that have adverse impacts on sexual well-being, including pain, vaginal dryness, sensations of burning, and irritation. These menopausal-related modifications involve a higher risk of vaginal and urinary infections, urinary leakage, vaginal tissue trauma, and quality of life impairment [4,5,6,7,8,9,10]. Additionally, vaginal relaxation syndrome may occur as a gradual weakening and laxity of the mucosa over time; this fact may lead to stress urinary incontinence, pelvic organ prolapse, sexual dysfunction, and other pelvic floor disorders [11,12,13,14].

GSM and VVA management depend on symptom severity, age, general health, and individual health risks [15]. The first-line treatment consists of lubricants, moisturizers, and local estrogen products. In recent years, newer hormonal therapeutic strategies have been proposed, including selective estrogen receptor modulators (SERMs). However, in survivors of breast or gynecological cancer, energy-based therapeutic approaches—for instance, through radiofrequency or laser devices—may be employed as an alternative. Energy-based vaginal rejuvenation modalities could offer an alternative approach to addressing GSM/VVA, as they have the potential to enhance vascularization and the overall integrity of connective tissue throughout the vaginal canal. This, in turn, may lead to the alleviation of symptoms associated with that condition [16,17]. Nowadays, multiple studies are available in the literature about the role of energy-based devices that may be safely and effectively offered to these patients.

Since the diagnosis of GSM relies solely on symptoms, the lack of standardization and objective and reproducible tools may lead to the insufficient monitoring of disease progression and treatment response. Understanding the characteristics of the vaginal wall is crucial to understanding its physiological functions, as well as for diagnosing and treating various conditions that may affect this anatomical structure and evaluating the efficacy of treatments. While histologic analysis provides an objective approach to evaluate this change, its application through biopsy is invasive and not conducive to standard clinical practice. Current non-biopsy objective measures for genitourinary syndrome of menopause (GSM) include the vaginal maturation index [18] and vaginal pH [19]; however, these methods exhibit weak correlations with patient-reported symptomatology. Weber [20] proposed the potential use of focal depth measurements of the vagina via the Cytocam-Incident Dark Field Device, but further research is imperative before its integration into clinical practice. Despite the existence of other subjective assessments for evaluating vaginal atrophy, the current approach emphasizes a comprehensive assessment that integrates multiple data points and clinical signs, encompassing the presence of vaginal symptoms, clinical examination findings, and cytologically determined atrophy [18].

Considering the widespread use, safety, and reliability of ultrasound in evaluating gynecological anatomy and pathological alterations, it presents a promising avenue for investigating variations in vaginal wall thickness [3,20,21].

Although multiple approaches have been described in the literature, a standard measurement technique is not yet universally accepted and routinely applied, and there have been no studies that have compared the use of all these techniques with each other. Consequently, a consensus regarding the optimal technique for measuring vaginal wall thickness (VWT) is still absent in the literature.

Despite numerous studies employing transabdominal convex probes and 2D or 3D high-frequency transvaginal ultrasound probes, there is a notable absence of research utilizing the transvaginal biplanar probe for VWT measurement. Specifically, the use of the endovaginal linear crystal offers some substantial advantages compared to all the other proposed methods. First of all, the structure which is aimed to be measured—the vaginal wall thickness—lies perpendicular to the sonographic beam, which maximizes the accuracy of the measurements. Moreover, contrary to all the other ultrasound approaches, in this case the probe is not curved, thus eliminating any kind of geometrical distortion.

Consequently, with this study, we aimed to validate a new method for measuring VWT using a linear transvaginal ultrasound probe, and assess both intra-operator and inter-operator reproducibility.

## 2. Material and Methods

This was a prospective study. Approval from the local Ethics Committee was obtained before the start of the study (protocol code: GSM-LASER). Recruitment occurred from September 2023 to December 2023 in the gynecologic outpatients in Fondazione IRCCS San Gerardo dei Tintori, Monza, Italy. Patients were sourced from individuals seeking care for GSM-related symptoms at the outpatient department of a tertiary referral center specializing in urogynecology. A detailed medical history was acquired, and each woman underwent a pelvic examination. The inclusion criteria involved a minimum age of 18 years with a diagnosis of GSM. Exclusion criteria included pregnancy, prior vaginal surgery, any connective tissue disorder impacting vaginal tissue, and inability to assume the lithotomy position due to musculoskeletal or mobility constraints.

**Figure 1:**
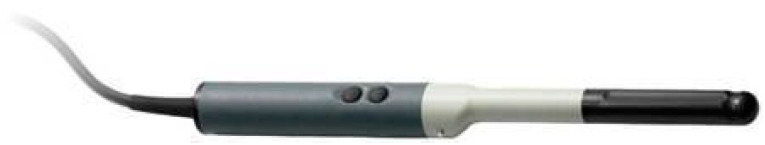
Endovaginal biplanar probe (BK Medical probe 8848, BK Ultrasound, Peabody, MA, USA).

Written consent was secured, and women underwent ultrasound scanning using a BK Medical Flex Focus 400 with an endovaginal biplanar probe (BK Medical probe 8848, BK Ultrasound, Peabody, MA, USA). The 65 × 5.5 mm linear longitudinal transducer was used to obtain midline scans of the anterior and posterior vaginal walls (Figure 1).

**Figure 2:**
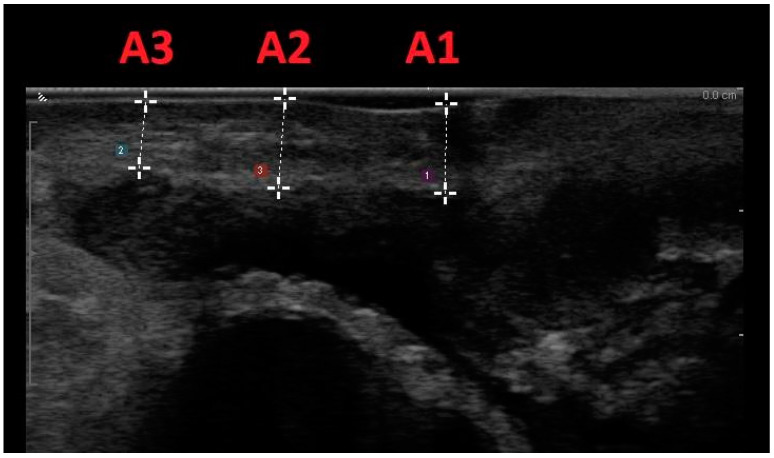
Midline sagittal scan of the anterior vaginal wall with transvaginal biplanar probe; the anatomical sites were at the level of the bladder neck (A1), in the anterior fornix (A3), and halfway between the two (A2).

**Figure 3:**
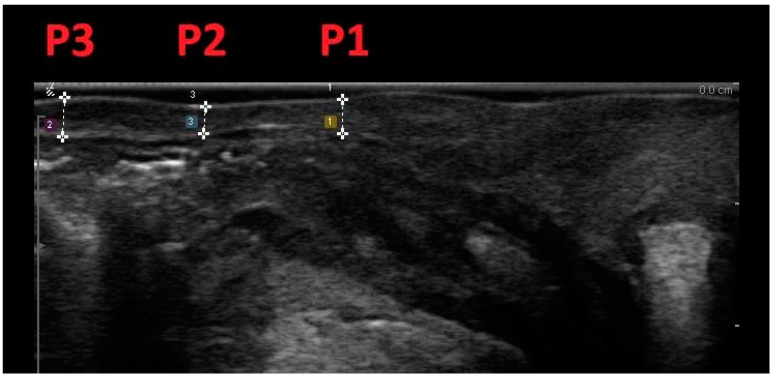
Midline sagittal scan of the posterior vaginal wall with transvaginal biplanar probe; vaginal wall thickness was measured at the level of the anorectal junction (P1), at the posterior fornix (P3), and halfway between the two (P2).

This methodology involved scanning women positioned in lithotomy at rest, within 15 min after bladder voiding, with ultrasound confirmation of a bladder volume less than 50 mL. Vaginal wall thickness (VWT) measurements were acquired at three anatomical points on the anterior vaginal wall and three locations on the posterior vaginal wall, utilizing a transvaginal scanner in the sagittal plane. Specifically, measurements on the anterior vaginal wall included the bladder neck level (A1), the anterior fornix (A3), and the midpoint between the two (A2). On the posterior vaginal wall, measurements were taken at the anorectal junction level (P1), the posterior fornix (P3), and the midpoint between the two (P2) (Figure 2 and Figure 3).

The transvaginal probe was gently applied to the vaginal wall with the minimum pressure necessary for contact, aiming to prevent any distortion or pressure-induced alterations to measurements. Vaginal wall thickness measurements were defined as the perpendicular thickness of the tissue between the transvaginal probe and the specified anatomical site. Calipers were positioned at the vaginal wall’s edge nearest to the probe and at the point closest to, but not in contact with, the adjacent organ at the relevant anatomical site. For instance, when measuring the vaginal wall thickness at the bladder neck on the anterior vaginal wall, the caliper would be placed at the vaginal wall’s edge closest to the ultrasound probe, and then at the point nearest to, but not in contact with, the bladder neck.

At the first visit, the patients underwent scanning at each of the six anatomical locations, by two different and blinded operators employing the technique outlined earlier. Subsequently, participants were requested to return after a 2-week interval, at which point they underwent a repeat transvaginal scan, conducted by the same single operator. In the given timespan (2 weeks), patients did not receive any treatment for GSM.

Anonymized data were entered into the database by the authors. Statistical analysis was performed using JMP software version 9 (SAS Institute, Cary, NC, USA). Outcomes were reported as mean ± standard deviation for continuous variables and as number (percentage) for noncontinuous variables. Reproducibility was evaluated using the intraclass correlation coefficient (ICC). The ICC was calculated for each measure for the intra-observer assessment and the inter-observer assessment. The range of variability between the two evaluations was calculated using the paired score differences of each test. The mean value and standard deviation (SD) of the score differences were then calculated, and the SD was used to describe the spread of score differences. The 95% confidence intervals of the mean difference are thus the boundaries of the expected range of variability.

## 3. Results

The two operators conducted scans on eleven women during the initial visit, and a single operator performed scans during a subsequent visit, following the described methods. The mean age of the participants was 68 years (range: 46–82 years), with a mean parity of two (range: 0–3).

Table 1 presents the mean and standard deviation of vaginal wall thickness measurements obtained by the two operators during the first visit, along with the mean difference and the 95% confidence interval of the mean difference (inter-observer reliability). Additionally, the 95% confidence interval of the mean difference, expressed as a percentage of the mean vaginal wall thickness, is provided.

The table indicates robust consistency between operators in vaginal wall thickness measurements across all vaginal sites, accompanied by a minimal mean difference between the operators. The intra-class coefficient ranged from 0.931 to 0.987, but at all anatomical sites, the value was higher than 0.9.

Table 2 presents the mean and standard deviation of vaginal wall thickness measurements conducted by a single operator during two distinct examinations, along with the mean difference between visits and the corresponding 95% confidence interval of the mean difference. The percentage variation in the 95% confidence interval from the mean is also depicted. The findings demonstrate robust consistency in vaginal wall thickness measurements obtained by a single operator during each visit, as indicated by low mean differences and intra-class coefficients exceeding 0.9 in each instance.

## 4. Discussion

As previously mentioned, genitourinary syndrome of menopause (GSM) and vulvovaginal atrophy (VVA) represent frequently reported menopause-related clinical entities [1].

Since the mucosal layer of the vagina is primarily composed of an estrogen-sensitive stratified squamous epithelium, the thickness of the vaginal wall decreases after menopause [3]. The reduced thickness of the vaginal epithelium can lead to vaginal atrophy, a condition marked by diverse symptoms that have adverse impacts on sexual well-being, including pain, vaginal dryness, sensations of burning, and irritation.

Understanding the characteristics of the vaginal wall is crucial to understanding its physiological functions, as well as for diagnosing and treating various conditions that may affect this anatomical structure and evaluating the efficacy of treatments. Specifically, the vaginal wall comprises multiple layers of tissue that confer support, elasticity, and protective functions. Histologically, the vagina is organized into four layers, extending from the lumen outward [22]:

- *Mucosa:* Comprising an estrogen-sensitive stratified squamous epithelium, the mucosa exhibits a multi-layered epithelium with three distinctive layers. These include the basal layer with cylindrical cells (germinative layer), overlying two or three layers of polyhedral cells (deep spinous layer). The intermediate layer consists of flattened cells, while the superficial layer comprises multiple rows of flattened cells. This specialized epithelium offers protection against the abrasion and friction associated with sexual activity and childbirth. Additionally, the mucosa may exhibit folds or rugae, facilitating expansion and contraction during various physiological activities.
- *Lamina Propria:* Situated beneath the epithelium, the lamina propria serves as a connective tissue layer providing support to the mucosal layer. Blood vessels, nerves, and lymphatic vessels within the lamina propria contribute to the vascularization and innervation of the vaginal wall.
- *Muscularis:* Comprising smooth muscle fibers arranged in an inner circular layer and an outer longitudinal layer, the muscularis layer enables the contraction and relaxation of the vaginal wall. This functionality supports various physiological functions, including sexual activity and childbirth.
- *Adventitia or Serosa:* Constituting the outermost layer of the vaginal wall, this layer consists of loose connective tissue referred to as adventitia. In specific regions, such as the upper portion of the vagina, a serosal layer may be present instead of adventitia. The adventitia/serosa provides structural support and aids in anchoring the vagina to surrounding structures, such as the pelvic floor [22].

Considering the widespread use, safety, and reliability of ultrasound in evaluating gynecological anatomy and pathological alterations, it presents a promising avenue for investigating variations in vaginal wall thickness [3,20,21]. In fact, pelvic ultrasound represents a non-invasive, reliable technique for obtaining gynecological measurements [21]. For instance, the assessment of endometrial lining through transvaginal ultrasound is a widely employed and validated method for evaluating postmenopausal uterine bleeding [23]. Another widespread application is the use of two-dimensional ultrasound to assess the bladder wall thickness. Initially introduced in 1994, this technique has undergone validation [24]. Subsequently, advancements have expanded its applicability, allowing measurement of the bladder wall through transabdominal, transvaginal, and transperineal approaches [25,26,27].

In this manuscript, we aimed to evaluate a novel methodology for the measurement of vaginal wall thickness utilizing a transvaginal biplanar ultrasound probe. For the measurement of vaginal wall thickness (VWT), various ultrasound techniques have been proposed in the literature [3,28,29,30,31,32,33,34,35], both transabdominal and transvaginal and both 2D and 3D at high frequency. Panayi first described a transvaginal US technique to measure the thickness of the vaginal wall at various anatomical sites, including the bladder neck, anterior and posterior fornix, and rectum [21]. Then, Balica proposed measuring VWT and total vaginal mucosa thickness using transabdominal sonography in the sagittal plane at the bladder trigone, with a full bladder approach [31]. Recently, Pereira compared VWT measurement using transabdominal and transvaginal approaches [33]. 

The measurements were conducted on the anterior and posterior vaginal walls, specifically at the proximal third of the vagina (including the anterior and posterior vaginal fornix), the middle third (at the junction between the proximal urethra and rectum), and the distal third (beyond the urethra/vaginal introitus and anorectal junction). Significant variations in vaginal wall thickness (up to 3 mm) were noted by these investigators, based on the chosen approach. This underscores the fact that the two techniques cannot be employed interchangeably [33]. Moreover, Peker [32] and Alcazar [29] proposed the use of two different methods of 3D transvaginal ultrasound for measuring VWT. Peker proposed measuring VWT anteriorly at the level of the bladder neck and posteriorly at the level of anorectal junction [32], while Alcazar proposed measuring VWT in the second axial plane, at a distance of 2 cm from the posterior vaginal fornix, at 12, 3, 6, and 9 o’clock [29]. Interestingly, the correlation between age, time from menopausal status, and sexual function, evaluated through FSFI-19, has been shown to be related to the sonographic measurement of vaginal thickness, suggesting a role of this parameter in the diagnosis and treatment efficacy monitoring of GSM [32]. To date, no previous study has validated VWT through a transvaginal biplanar ultrasound probe. Two previous studies evaluated VWT measurement with a transvaginal linear probe in patients with pelvic organ prolapse [36,37]. Manodoro et al. evaluated, with an endovaginal linear probe, the thickness of pubocervical fascia in patients with an anterior compartment central defect, compared to patients with other defects. They found that the thickness of the pubocervical fascia was significantly lower in patients with a central defect compared to controls [36]. Barba et al., with the same probe, compared the ultrasonographic thickness of the rectovaginal septum before and after native-tissue surgical repair, either including posterior colporrhaphy or not. They demonstrated an increased thickness only in patients who underwent posterior colporrhaphy during surgery [37]. However, in both papers, the method had not previously been standardized.

Panayi was the first to propose VWT ultrasound measurement using the transvaginal route [21]. This author advocated for the measurement of vaginal wall thickness in the sagittal plane, specifically targeting three anatomical landmarks on the anterior vaginal wall (bladder neck, apex of the bladder, and anterior fornix) and three sites on the posterior vaginal wall (the anorectal junction, the rectum, and the posterior fornix). In contrast to Panayi’s approach, our study introduced anatomical landmarks positioned at different locations. Specifically, our proposed landmarks were identified on the anterior vaginal wall at the level of the bladder neck (A1), within the anterior fornix (A3), equidistant between the two (A2), and on the posterior vaginal wall at the level of the anorectal junction (P1), within the posterior fornix (P3), and equidistant between the two (P2). As in our study, Panayi proposed applying the transvaginal probe on the vaginal wall with minimal pressure to prevent any distortion or pressure-related effects on ultrasound findings [21].

Pereira subsequently conducted a comparative analysis of vaginal wall thickness measurements using transabdominal and transvaginal approaches [33]. The anatomical landmarks proposed were the same ones used in our study. However, unlike our study, in the transvaginal approach they used 40 mL of water-based gel to separate the vaginal walls and avoid pressure on the vaginal walls by the probe. Significant differences in vaginal wall thickness (up to 3 mm) were observed between the two techniques, indicating that the two techniques were not interchangeable [33].

More recently, Ros [35] conducted a study following a transvaginal approach, with vaginal distension like Pereira’s method [33], but measuring vaginal wall thickness in the four quadrants, addressing not only the anterior and posterior vaginal walls but also the right and left lateral walls. Measurements were taken in the proximal third of the vagina, “near the cervix”, although the specific measurement locations were not specified [35].

The use of the transvaginal probe was also proposed by Peker who, however, preferred the use of a high-resolution 3D endovaginal–endoanal ultrasound probe for measuring VWT [32]. Measurements were taken at the bladder neck and anorectal junction. His method involved inserting the probe into the vagina without gel.

Subsequently, Alcazar proposed measuring VWT using three-dimensional ultrasound, after filling the vagina with gel. The measurement of vaginal wall thickness was performed at a distance of 2 cm from the posterior vaginal fornix at 12, 3, 6, and 9 o’clock [29], as previously proposed by Ros [35].

The objective of this study was to standardize the technique for measuring VWT through the use of biplane transvaginal ultrasound. The validation of a method can be established by either demonstrating its effectiveness compared to a gold standard or by confirming the repeatability of the technique. Since there is currently no gold standard for the non-invasive measurement of VWT, for the validation of our measurement system it was necessary to evaluate its intra-operator and inter-operator repeatability. We found that the transvaginal linear ultrasound measurement of VWT proved to be a reliable method for measuring vaginal wall thickness, with excellent inter-observer and intra-observer reliability, establishing its validity as a clinical tool.

Despite the limited sample size, our preliminary findings indicate the feasibility of the proposed approach, showcasing its applicability across all patients and the reproducibility of measurements. In fact, for each measurement taken at the six anatomical sites, three on both the anterior and posterior vaginal walls, the intra-class correlation coefficient (ICC) exceeded 0.9, indicating strong inter-operator and intra-operator agreement. Specifically, the intra-operator data exhibited excellent repeatability, consistently attaining an intra-class correlation coefficient (ICC) greater than 0.95 at all vaginal sites. Although the intra-operator agreement showed ICC values lower than the intra-observer agreement across all vaginal sites, the inter-observer repeatability remained strong, consistently exceeding 0.9. The variations in the mean differences observed in the intra- and inter-observer data across different anatomical sites remained negligible.

Panayi also reported similar findings in terms of both inter-operator and intra-operator repeatability [10]. In his study, even without using gel to distend the vagina, excellent results were reported for intra-operator repeatability, and good results for inter-operator repeatability, when measuring VWT at three different anatomical landmarks of the anterior and posterior vaginal walls. Concerning other studies that have introduced methodologies for measuring VWT using a transvaginal ultrasound probe with vaginal gel filling, such as those by Ros [35] or Pereira [33], the evaluation of inter-operator and intra-operator repeatability was not addressed. Also, Peker’s study, which investigated the use of the 3D transvaginal probe without vaginal gel filling, did not investigate intra-operator or inter-operator variability [32]. With respect to the application of 3D transvaginal ultrasound for measuring VWT following vaginal gel filling, Alcazar conducted a study specifically focusing on the inter-operator repeatability of measurements at the four vaginal fornices. The results of his study indicated good inter-operator repeatability [29], comparable to the inter-operator repeatability described in our study. For this reason, it can be asserted that the distortion effect attributed to the pressure applied by the transvaginal probe, which might be a potential factor influencing inter-operator variability in our study, is minimal.

Our method also facilitated the acquisition of measurements, as evidenced by our finding that neither operator encountered challenges in measuring the thickness of the vaginal wall at each patient’s vaginal site. Each operator successfully obtained measurements without encountering any instances of missing values. Our investigation demonstrated the applicability of transvaginal biplanar ultrasound for measuring VWT across all women. The technique’s ease of acquisition and swift execution make it well-suited for application in outpatient settings. Panayi [21] and Alcazar [29] also reported similar results regarding the feasibility of ultrasound in each patient. In their studies, each operator successfully obtained measurements of VWT using the technique proposed in their respective investigations.

Our proposal to employ transvaginal biplanar ultrasound holds potential for the precise identification of anatomical landmarks, facilitating standardized and reproducible measurements. Specifically, the use of the endovaginal linear crystal offers some substantial advantages compared to all the other proposed methods. First of all, the vaginal wall thickness measurement results are perpendicular to the sonographic beam, which maximizes the accuracy of the evaluation. Moreover, contrary to all the other ultrasound approaches, in this case the probe is not curved, thus eliminating any kind of geometrical distortion. Lastly, the probe encloses 65 mm of the scan, usually enough to evaluate the whole region of interest. However, our study has some limitations, including the limited population, and the lack of widespread diffusion of biplanar transvaginal probes, which are not available in all ultrasound equipment.

Given the good repeatability results achieved with the proposed technique in this study, it becomes intriguing to explore the possibility of establishing standard measurements in “healthy vaginas” to provide a valuable benchmark for comparing pathological conditions associated with variations in vaginal thickness. Additionally, having an ideal measurement for healthy individuals could serve as a reference for evaluating the efficacy of therapies designed to address these conditions.

## 5. Conclusions

Transvaginal biplanar ultrasound measurement proved to be a reliable method for measuring vaginal wall thickness. In this pilot study, the suggested approach exhibited good inter-observer and intra-observer reliability, establishing its validity. Consequently, this technique holds promise for inclusion in larger-scale studies on vaginal wall measurements.

## Figures and Tables

**Table 1:** Inter-observer reliability: illustrated by presenting the mean and standard deviation of vaginal wall thickness measured by the two operators during the first examination. The analysis includes the mean difference, standard deviation of the mean difference, the 95% confidence interval of the mean difference, and the intra-class coefficient.

Vaginal Points	First Operator Mean Measure of VWT in mm (SD)	Second Operator Mean Measure of VWT in mm (SD)	Mean Difference in mm	Standard Error in mm of the Mean Difference	95% IC in mm	ICC
A1	2.8 (1.49)	3.0 (1.08)	−0.16	0.18	−0.56/0.23	0.945
A2	2.7 (1.64)	2.8 (1.42)	0.05	0.1	−0.17/0.27	0.987
A3	2.7 (1.39)	2.7 (1.29)	0.08	0.15	−0.26/0.42	0.931
P1	2.8 (1.95)	3.4 (1.94)	−0.06	0.19	−0.47/0.34	0.952
P2	3.0 (1.26)	2.8 (1.23)	0.05	0.08	−0.14/0.23	0.975
P3	2.3 (1.46)	2.7 (1.62)	0.18	0.12	−0.09/0.46	0.974

**Table 2:** Intra-observer reliability was assessed by presenting the mean and standard deviation of vaginal wall thickness measurements conducted by operator 1 during two distinct examinations. Additionally, the 95% confidence interval of the mean difference between the two visits, the difference, and the intra-class coefficient are included in the analysis.

Vaginal Points	First Operator Mean Measure of VWT in mm (SD) at 1st Visit	First Operator Mean Measure of VWT in mm (SD) at 2nd Visit	Mean Difference in mm	Standard Error in mm of the Mean Difference	95% IC in mm	ICC
A1	2.8 (1.49)	3.0 (1.33)	−0.03	0.10	−0.26/0.19	0.977
A2	2.7 (1.64)	3.0 (1.39)	0.02	0.09	−0.20/0.24	0.989
A3	2.7 (1.39)	2.6 (1.23)	−0.01	0.11	−0.24/0.23	0.972
P1	2.8 (1.95)	3.2 (1.74)	0.05	0.10	−0.18/0.29	0.988
P2	3.0 (1.26)	3 (1.01)	0.10	0.11	−0.14/0.34	0.976
P3	2.3 (1.46)	2.5 (1.49)	0.08	0.13	−0.20/0.36	0.980

## Data Availability

The data presented in this study are available on request from the corresponding author.
